# Supplementary material for: Establishment and verification of a nomogram model for predicting the risk of post-stroke depression
Source: PeerJ. 2023 Feb 2;11:e14822. doi: 10.7717/peerj.14822 (PMC9899426; doi:10.7717/peerj.14822)
Supplement: Supplemental Information 5 [file peerj-11-14822-s005.docx]

1) Sex: ① male; ② female

2) Age: ① ≥ 60 years old; ② < 60 years old

3) Education level: ① primary school or below; ② junior high school or senior high school; ③ college or junior college or above

4) Occupation: ① farmer; ② civil servant or staff; ③ service industry; ④ others

5) Working status: ① full-time; ② retirement; ③ others

6) The mode of work: ① physical labor; ② mental labor; ③ physical labor is equal to mental labor

7) Marital status: ① married; ② unmarried; ③ divorced or widowed

8) Number of children: ① 0; ② 1; ③ 2 or more

9) Smoking history: ① quit smoking; ② less than 20 cigarettes per day; ③ more than 20 cigarettes per day; ④ no smoking

10) Drinking history: ①drinking; ② a small amount; ③ drinking more than 50g per day

11) Diabetes history: ① none; ② but with poor or untreated treatment; ③ with good treatment; ④ do not know whether there is a history of diabetes

12) History of hypertension: ① none; ② with poor treatment or without treatment; ③ with good treatment; ④ do not know whether there is such a history

13) History of hyperlipidemia: ① none; ② yes

14) TIA history: ① none; ② yes

15) The history of operation: ① none; ② yes

16) History of interest and love: ① none; ②yes

17) The time from onset to hospitalization: ① less than or equal to 3 days; ② > 3 days less than or equal to 7 days; ③ > 7 days less than or equal to 11 days; ④ > 11 days.

18) Stroke area: Patients received an MRI examination after admission, and the stroke area was classified according to: ①anterior circulation; ② posterior circulation; ③anterior and posterior circulation are involved.

19) NIHSS:: ①0–1 point; ②1–4 points; ③5–15 points
